# The Cl^−^ transporter ClC-7 is essential for phagocytic clearance by microglia

**DOI:** 10.1242/jcs.261616

**Published:** 2024-02-16

**Authors:** Harini Iyer, William S. Talbot

**Affiliations:** Department of Developmental Biology, Stanford University School of Medicine, Stanford, CA 94305, USA

**Keywords:** ClC-7, Ion channel, Macrophages, Microglia, Ostm1, Zebrafish

## Abstract

Microglia, professional phagocytic cells of the brain, rely upon the appropriate activation of lysosomes to execute their immune and clearance functions. Lysosomal activity is, in turn, modulated by a complex network of over 200 membrane and accessory proteins that relay extracellular cues to these key degradation centers. The ClC-7 chloride (Cl^−^)-proton (H^+^) antiporter (also known as CLCN7) is localized to the endolysosomal compartments and mutations in *CLCN7* lead to osteopetrosis and neurodegeneration. Although the functions of ClC-7 have been extensively investigated in osteoclasts and neurons, its role in microglia *in vivo* remains largely unexamined. Here, we show that microglia and embryonic macrophages in zebrafish *clcn7* mutants cannot effectively process extracellular debris in the form of apoptotic cells and β-amyloid. Despite these functional defects, microglia develop normally in *clcn7* mutants and display normal expression of endosomal and lysosomal markers. We also find that mutants for *ostm1*, which encodes the β-subunit of ClC-7, have a phenotype that is strikingly similar to that of *clcn7* mutants. Together, our observations uncover a previously unappreciated role of ClC-7 in microglia and contribute to the understanding of the neurodegenerative phenotypes that accompany mutations in this channel.

## Teaser

**Highlighted Article:** Microglia, brain-resident macrophages, require the endolysosomal Cl^−^ transporter ClC-7 for effective clearance of debris.

## INTRODUCTION

Ion channels and transporters play a pivotal role in maintaining cellular and tissue homeostasis by controlling osmosis and cell volume, maintaining the appropriate pH of cellular organelles, and regulating membrane potential (Li et al., 2019). Lysosomes, as nutrient and stress sensors of the cell, especially rely on ion channels and transporters to transduce rapidly changing environmental and metabolic signals (Kendall and Holian, 2021). Furthermore, as highly acidic organelles of the cell, lysosomes and lysosomal activity are acutely sensitive to fluctuations in the ion balance (Riederer et al., 2023). The importance of ion flux for lysosomal function is underscored by observations showing that disrupted activity of ion channels and transporters leads to defects in degradation and recycling by lysosomes (Kendall and Holian, 2021). For example, mucolipidosis type IV, a lysosomal storage disorder, results from mutations in the Ca^2+^ channel *TRPML1* (Dong et al., 2008). In addition, mutations in the vacuolar-H^+^-ATPase (*TCIRG1*) and chloride channel-7 (*CLC-7*; also known as *CLCN7*) lead to autosomal recessive osteopetrosis due to aberrant lysosomal activity in osteoclasts and consequent defects in bone resorption (Kornak et al., 2001; Susani et al., 2004).

Microglia, as the primary phagocytic cells of the central nervous system, depend on lysosomal activity to execute their surveillance and clearance functions (Holtman et al., 2015; Majumdar et al., 2007; Plaza-Zabala et al., 2017; Sole-Domenech et al., 2016). In addition, core microglial activities, including proliferation, migration, phagocytosis and release of cytokines depend on the proper regulation of ion flux (Kettenmann et al., 2011). For instance, Ca^2+^ transporters in microglia influence migration, ramified morphology and podosome formation in these cells; Na^+^ and K^+^ channels direct migration, volume regulation and reactive oxygen species production by microglia; and Cl^−^ channels influence the membrane potential, phagocytosis, filopodia formation and production of inflammatory cytokines by microglia (Furtner et al., 2007; Hines et al., 2009; Milton et al., 2008; Newell and Schlichter, 2005; Novarino et al., 2004). Aberrant expression of these channels in microglia has been implicated in neuropathic pain, ischemic stroke, traumatic brain injury and, most commonly, in neurodegenerative conditions associated with aging, including Alzheimer's disease and Parkinson's disease (Izquierdo et al., 2019).

Intracellular Cl^−^ levels are tightly regulated by Cl^−^ channels, including cystic fibrosis transmembrane regulator (CFTR) and the CLC family of chloride channels and Cl^−^–H^+^ antiporters (Chakraborty et al., 2017). CLC proteins have vital roles in regulating electrical excitability and maintaining the lumenal pH of subcellular compartments (Bose et al., 2021). Furthermore, these proteins localize to distinct compartments of the endolysosomal system, and mutations in the *CLC* family lead to lysosomal storage and processing defects (Chen et al., 2020; Pressey et al., 2010). In addition, mutations in *Clcn7*, and its β-subunit *Ostm1*, also lead to osteopetrosis, severe retinal degeneration and neurodegeneration (Kasper et al., 2005). Although the functions of ClC-7 and Ostm1 have been widely investigated in osteoclasts (bone macrophages) and neurons (Bose et al., 2021; Kornak et al., 2001), their functions in microglia during development remain largely unexamined. Here, we show that microglia display an inability to effectively clear apoptotic and neuronal debris in zebrafish *clcn7* mutants. In addition, embryonic macrophages and microglia in *clcn7* mutants are unable to process exogenous substrates, including bacteria and β-amyloid peptide (1–42). Despite these functional deficits, microglia and embryonic macrophages develop normally in *clcn7* mutants, display normal expression of the markers of late endosomes and lysosomes, and respond appropriately to a variety of chemotactic cues. Our results uncover the role of ClC-7 in microglia and provide a new understanding of ClC-7-mediated neurodegeneration.

## RESULTS AND DISCUSSION

### Microglia in *clcn7* and *ostm1* mutants show reduced accumulation of Neutral Red

**Fig. 1.**
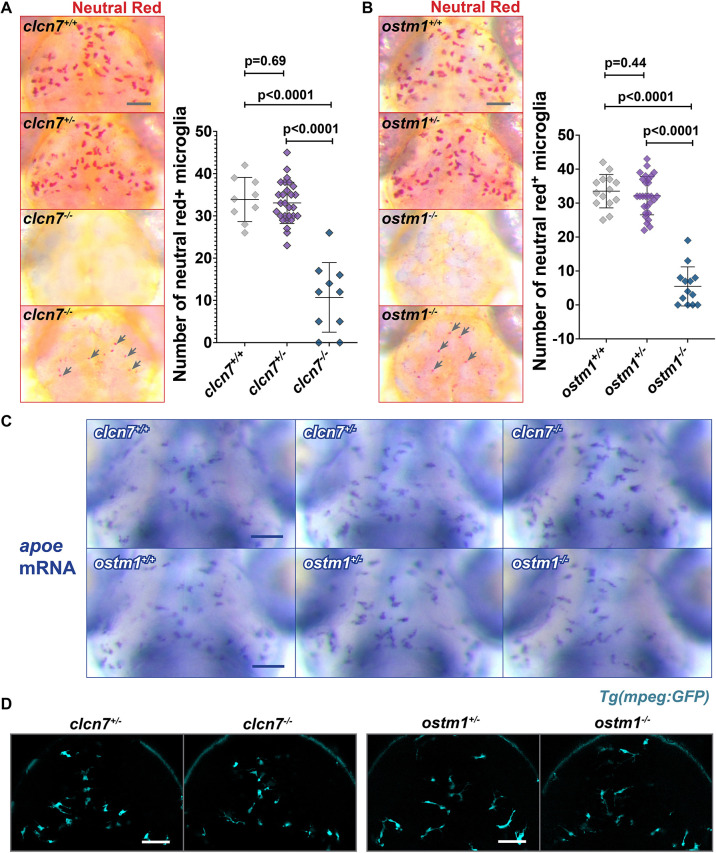
**Reduced Neutral Red staining in *clcn7* and *ostm1* mutants.** (A,B) Neutral Red labeling revealed significantly fewer microglia labeled with this vital dye in (A) *clcn7* and (B) *ostm1* mutants. Some mutants displayed small Neutral Red punctae (gray arrows). These smaller Neutral Red punctae were included in the quantification. *clcn7*^+/+^: *n*=9, mean=33.9, s.d.=5.2, s.e.m.=1.7. *clcn7*^+/−^: *n*=28, mean=33.1, s.d.=4.9, s.e.m.=1.0. *clcn7*^−/−^: *n*=10, mean=10.7, s.d.=8.2, s.e.m.=2.6. *ostm1*^+/+^: *n*=14, mean=33.5, s.d.=4.9, s.e.m.=1.3. *ostm1*^+/−^: *n*=28, mean=32.2, s.d.=5.6, s.e.m.=1.1. *ostm1*^−/−^: *n*=13, mean=5.5, s.d.=5.8, s.e.m.=1.6. Two-tailed unpaired *t*-test with Welch's correction was performed to calculate significance; graphs show mean±s.d. (C) *apoe in situ* hybridization showed that *clcn7* and *ostm1* mutants have normal numbers and morphology of microglia. (D) Confocal microscopy confirmed that *mpeg1:GFP* expression is normal in *clcn7* and *ostm1* mutants. Images in A, B and D are representative of at least three independent experiments, and those in C of two independent experiments. Scale bars: 50 µm.

To examine the functions of *clcn7* and *ostm1* in microglia, we generated zebrafish mutants for these genes using CRISPR-Cas9. The *clcn7* mutants (*st167*) carry a 22-bp deletion in exon 1, leading to an early stop codon in the coding sequence (Fig. S1A). The *ostm1* mutation (*st168*) is a 5-bp deletion in exon 5 (Fig. S1B). Neutral Red is a vital dye that is widely used to label microglia in zebrafish (Herbomel et al., 2001). We found that microglia in both *clcn7* and *ostm1* mutants showed sparse Neutral Red labeling (Fig. 1A,B). Notably, some *clcn7* and *ostm1* mutants displayed small Neutral Red punctae in the midbrain (Fig. 1A,B). These mutant phenotypes co-segregated with their respective lesions (numbers given in the legend to Fig. 1). In addition, co-injection of Cas9 and three different sgRNAs targeting other genomic regions in the *clcn7* locus caused a similar reduction in Neutral Red labeling in F0 CRISPR-injected animals (Fig. S2A). Neutral Red dye is red below pH 6.8 and yellow between 6.8 and 8.0, so the disruption in *clcn7* and *ostm1* mutants could reflect changes in pH or the loss of microglia.

To establish whether microglia are present in *clcn7* and *ostm1* mutants, we used alternative methods for labeling microglia. Previous research in zebrafish has shown that microglia express *apoeb* in microglia starting at ∼3 days post fertilization (dpf) (Peri and Nusslein-Volhard, 2008). Using *in situ* hybridization for *apoeb*, we found that *clcn7* and *ostm1* mutants display normal numbers and morphology of microglia (Fig. 1C). ClC-7 is a known target of the microphthalmia-associated transcription factor (MiTF) family, and members of this protein family have gene dosage-dependent effects (Iyer et al., 2022). However, we found that microglia in *clcn7* and *ostm1* heterozygotes appeared to be similar to microglia in the wild-type siblings as assessed by both the Neutral Red assay and *apoeb in situ* hybridization (Fig. 1A–C). We also used the *mpeg1:GFP* transgene, which drives GFP expression in cells of the macrophage lineage (Ellett et al., 2011), to visualize both microglia and embryonic macrophages. We found that cells of the macrophage lineage, including microglia (Fig. 1D) and peripheral macrophages in the head, yolk and tail regions (Fig. S2B), appeared normal in *clcn7* mutant larvae. Humans carrying a gain-of-function mutation in *CLCN7* show delayed myelination in the corpus callosum (Nicoli et al., 2019). To examine a marker of myelinating glia, we performed *in situ* hybridization for *myelin basic protein* (*mbp*) and found that *mbp* expression in *clcn7* and *ostm1* mutants appeared to be comparable to that in heterozygotes (Fig. S2C). Thus, mutations in *clcn7* and *ostm1* lead to strikingly similar phenotypes with reduced accumulation of Neutral Red in microglia, which could indicate a disruption in microglial pH or efferocytosis (Demy et al., 2020).

### Markers of late endosomes and lysosomes appear normal in *clcn7* mutants

**Fig. 2.**
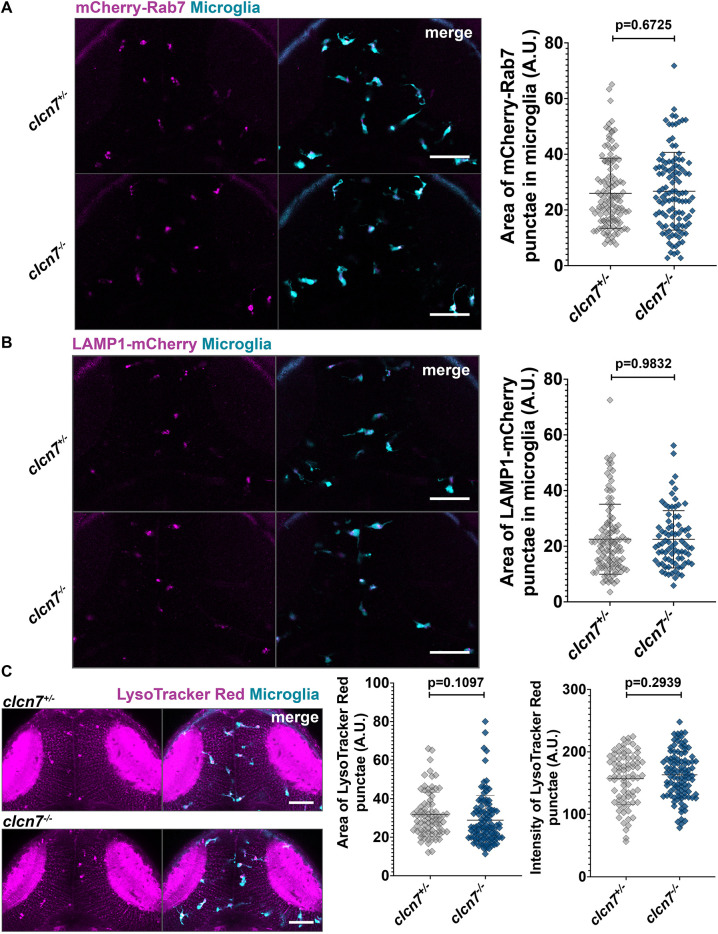
**Late endosomal and lysosomal compartments appear normal in *clcn7* mutants.** Transgenic expression of (A) *mCherry-Rab7* and (B) *LAMP1b-mCherry* driven under the control of *mpeg1* regulatory sequences was similar in *clcn7* heterozygotes and mutants. *mCherry-Rab7; clcn7^+/−^*: *n*=17, mean=26.0, s.d.=12.6, s.e.m.=1.1. *mCherry-Rab7; clcn7^−/−^*: *n*=8, mean=26.7, s.d.=14.0, s.e.m.=1.3. *LAMP1b-mCherry; clcn7^+/−^*: *n*=18, mean=22.5, s.d.=12.6, s.e.m.=1.2. *LAMP1b-mCherry; clcn7^−/−^*: *n*=8, mean=22.5, s.d.=10.3, s.e.m.=1.2. (C) Area and intensity of LysoTracker Red were similar between *clcn7* heterozygotes and mutants. *clcn7^+/−^* area: *n*=14, mean=31.8, s.d.=11.6, s.e.m.=1.3. *clcn7^−/−^* area: *n*=17, mean=28.8, s.d.=12.8, s.e.m.=1.3. *clcn7^+/−^* intensity: *n*=14, mean=157, s.d.=41.1, s.e.m.=4.7. *clcn7^−/−^* intensity: *n*=17, mean=163.3, s.d.=37.5, s.e.m.=3.7. Two-tailed unpaired *t*-test with Welch's correction was performed to calculate significance; graphs show mean±s.d. Images are representative of two independent experiments. Scale bars: 50 µm. A.U., arbitrary units.

Having established that mutants lacking ClC-7 and Ostm1 have similar phenotypes in microglia, we decided to focus on *clcn7* mutants to elucidate the functions of this ion transporter in cells of the macrophage lineage. Although ubiquitously expressed, ClC-7 is the only member of the ClC family that localizes to late endosomes and lysosomes (Brandt and Jentsch, 1995). Furthermore, other groups have shown that mutation in *Clcn7* can lead to the accumulation of electron-dense cytoplasmic material in neurons resembling lipofuscinosis (Kasper et al., 2005), suggesting possible defects in the endolysosomal pathway. To examine late endosomes and lysosomes in *clcn7* mutants, we used the previously generated transgenic zebrafish lines *mpeg1:mCherry-Rab7* and *mpeg1:LAMP1-mCherry* (Iyer et al., 2022). These fusion proteins, driven by the *mpeg1* regulatory sequences, label late endosomes and lysosomes in cells of the macrophage lineage respectively. Our live microscopy analysis revealed that expression of both mCherry–Rab7 and LAMP1–mCherry appeared to be comparable across *clcn7* mutants and controls (Fig. 2A,B), indicating that these fusion proteins are normally expressed in *clcn7* mutants. To further investigate lysosomes in *clcn7* mutants, we used LysoTracker Red, which is a dye that accumulates in acidic compartments of cells and has previously been used to label lysosomal compartments of microglia in zebrafish (Shen et al., 2016). We found that both the area and intensity of LysoTracker Red punctae were normal in *clcn7* mutants (Fig. 2C). Consistent with our above observations that the loss of *ostm1* leads to a phenotype strikingly similar to that of *clcn7* mutants, we found that *ostm1* mutants also exhibited normal staining by LysoTracker Red (Fig. S3A). Together, these observations demonstrate that markers of late endosomes and lysosomes appear to be normal in *clcn7* mutants.

### Microglia in *clcn7* mutants are unable to effectively degrade endogenous debris

**Fig. 3.**
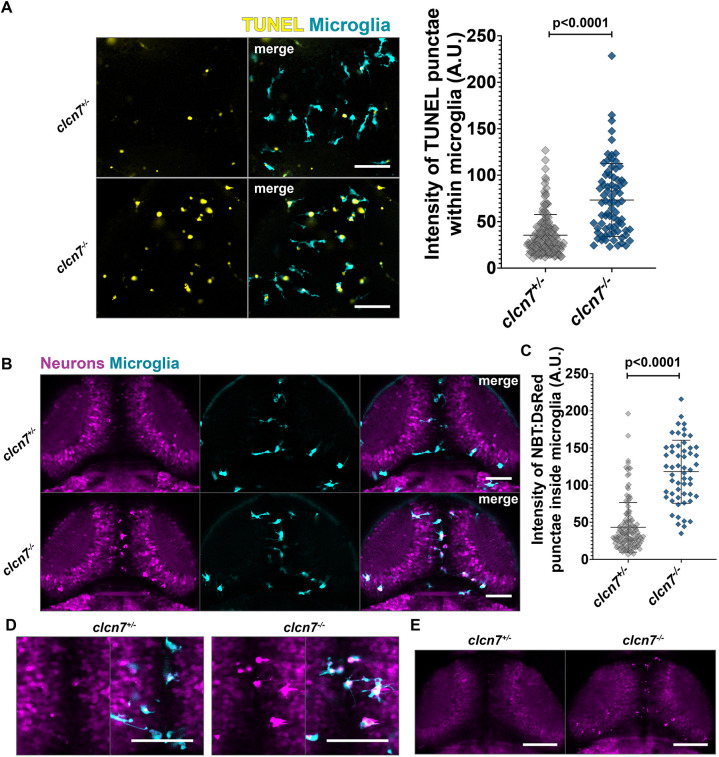
**Microglia are unable to effectively clear endogenous debris in *clcn7* mutants.** (A) TUNEL assay revealing a significantly greater intensity of apoptotic punctae inside the microglia of *clcn7* mutants (*n*=6, mean=73.3, s.d.=39.2, s.e.m.=4.5) than in *clcn7* heterozygotes (*n*=12, mean=35.5, s.d.=22.2, s.e.m.=1.8). (B) Microglia in *clcn7* mutants show an accumulation of NBT:DsRed neuronal punctae. (C) The intensity of DsRed punctae inside microglia was significantly greater in *clcn7* mutants (*n*=13, mean=118, s.d.=42.4, s.e.m.=5.7) relative to *clcn7* heterozygotes (*n*=29, mean=43.2, s.d.=33.4, s.e.m.=2.9). (D) High magnification image showing accumulation of neuronal debris inside microglia. (E) Low magnification image showing accumulation of NBT:DsRed punctae in the midbrain, where microglia are enriched. Images are representative of at least three independent experiments. Two-tailed unpaired *t*-test with Welch's correction was performed to calculate the significance in all graphs; graphs show mean±s.d. Scale bars: 50 µm. A.U., arbitrary units.

It was recently shown that ClC-7 is required for degradation by phagolysosomes in bone marrow-derived macrophages (Wu et al., 2023). As the primary phagocytic cells of the central nervous system, microglia perform several functions that are crucial for brain homeostasis and function, including clearing apoptotic debris, keeping neuronal progenitor numbers in check and eliminating misfolded proteins (Matcovitch-Natan et al., 2016; Sierra et al., 2010; Tremblay et al., 2011; Zhan et al., 2014). We proceeded to systematically examine each of these functions of microglia in *clcn7* mutants. First, we performed a TUNEL assay to visualize apoptotic debris clearance in the brain. Whereas *clcn7* heterozygotes had some TUNEL^+^ punctae inside microglia, the intensity of TUNEL^+^ punctae colocalizing with microglia was significantly greater in *clcn7* mutants (Fig. 3A). This observation indicates that microglia in *clcn7* mutants can effectively detect and ingest apoptotic debris but are apparently unable to effectively degrade the ingested apoptotic material. In addition to the TUNEL assay, we performed Acridine Orange staining (Tucker and Lardelli, 2007) and found that the numbers of Acridine Orange punctae in the head, eye and tail regions of *clcn7* mutants were comparable to the numbers found in wild-type siblings (Fig. S3B), thus indicating that global apoptosis was normal in *clcn7* mutants.

In addition to their role as scavengers of apoptotic debris, microglia also regulate the number of neural precursor cells and sculpt neural circuits by pruning synapses (Cunningham et al., 2013; Hoshiko et al., 2012; Miyamoto et al., 2016; Pont-Lezica et al., 2014; Schafer et al., 2012; Squarzoni et al., 2014). We used the *NBT:DsRed* transgene to visualize the uptake and clearance of neuronal debris by microglia via live imaging. The analysis revealed a marked accumulation of neuronal debris inside the microglia of *clcn7* mutants (Fig. 3B–E). Once again, to account for off-target effects, we used a combination of guide RNAs targeting three additional loci in the coding sequence of *clcn7* to confirm that microglia are unable to effectively process neuronal debris particles in *clcn7* CRISPR-injected F0 animals (Fig. S4A). To test the extent to which embryonic macrophages in *clcn7* mutants can effectively respond to a variety of chemotactic cues, we performed tail fin injury of zebrafish larvae and examined the macrophage response. Following injury, the macrophage response follows the early migration of neutrophils to the wound site (Mathias et al., 2009). We therefore examined the presence of macrophages at the wound site at 12 h post injury. We found that macrophages in *clcn7* wild-type siblings, heterozygotes and mutants exhibited similar responses to injury (Fig. S4B), indicating that macrophages in *clcn7* can effectively sense injury cues. Collectively, these observations establish that microglia in *clcn7* mutants can effectively sense both apoptotic and live cellular debris but are unable to degrade the ingested material.

### Exogenous debris is not efficiently cleared by embryonic macrophages and microglia in *clcn7* mutants

**Fig. 4.**
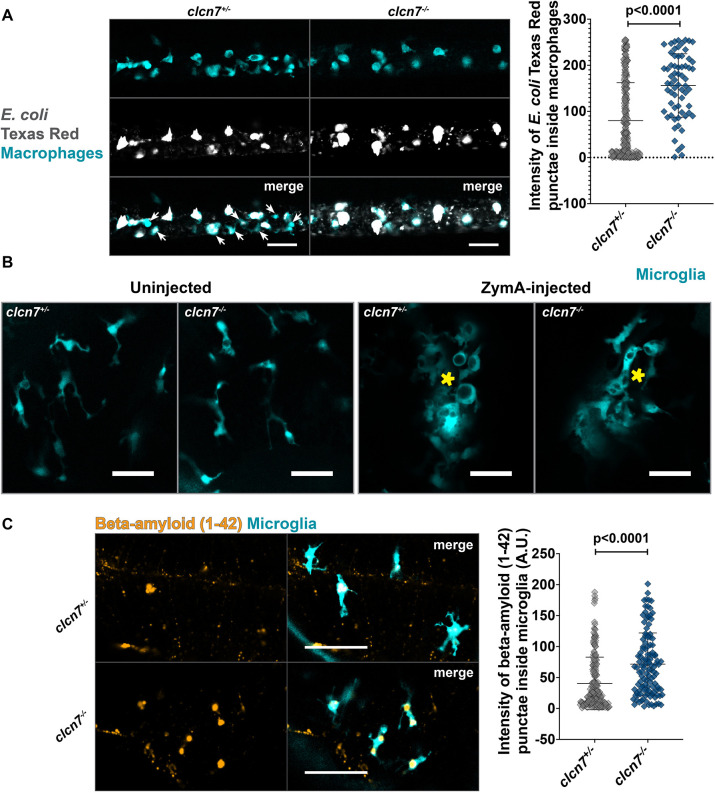
**Exogenous debris accumulates inside microglia and macrophages of *clcn7* mutants.** (A) *E. coli* Texas Red particles injected in the ducts of Cuvier accumulate inside peripheral macrophages. The intensity of Texas Red particles inside the macrophages is greater in *clcn7* mutants (*n*=8, mean=156.2, s.d.=68.8, s.e.m.=8.5) than in *clcn7* heterozygotes (*n*=27, mean=80.2, s.d.=82.3, s.e.m.=5.1). Arrows indicate macrophages containing little or no *E. coli* in control animals. Scale bars: 50 µm. (B) Zymosan A injected into the midbrain of zebrafish larvae to observe microglia response. Microglia in *clcn7* mutants and heterozygotes respond similarly to inflammatory stress. Yellow asterisks mark the site of injection. Images are representative of two independent experiments. Scale bars: 20 µm. (C) β-amyloid (1–42) HiLyte Fluor-555 particles injected into the midbrain of zebrafish larvae accumulate inside the microglia of *clcn7* mutants (*n*=16, mean=71.7, s.d.=50.3, s.e.m.=4.6) to a significantly greater extent than in *clcn7* heterozygotes (*n*=26, mean=40.5, s.d.=42.5, s.e.m.=3.0). Scale bars: 50 µm. Two-tailed unpaired *t*-test with Welch's correction was performed to calculate the significance in all graphs; graphs show mean±s.d.

Next, we wanted to test the ability of embryonic macrophages and microglia to degrade exogenous substrates in *clcn7* mutants. First, we challenged peripheral embryonic macrophages by injecting *Escherichia coli* Texas Red microbial debris particles into the ducts of Cuvier of zebrafish larvae (Fig. 4A). Macrophages in both *clcn7* heterozygotes and mutants actively phagocytosed *E. coli* particles and became activated and amoeboid in the process. However, the intensity of Texas Red punctae inside the macrophages of *clcn7* mutants was significantly greater compared to that in *clcn7* heterozygotes (Fig. 4A), indicating a greater accumulation of microbial debris inside the macrophages of *clcn7* mutants relative to heterozygotes. Next, to induce sterile inflammation, we injected Zymosan A into the midbrain ventricles of zebrafish larvae and found that the microglial response to inflammatory stress in *clcn7* mutants was similar to that in the heterozygotes (Fig. 4B). It has been previously reported that 1-phenyl 2-thiourea (PTU) treatment leads to elevated autophagy in zebrafish larvae (Chen et al., 2021). In addition to ensuring that wild-type, heterozygotes and mutant larvae were maintained under identical conditions in all our experiments, we also performed Zymosan A injection into the ducts of Cuvier in larvae not treated with PTU. We chose to examine the peripheral innate immune response because, in the absence of PTU treatment, the high density of pigment cells in the dorsal skin hinders the visualization of microglia. We found that the response of macrophages in *clcn7* mutants was comparable to that of controls (Fig. S4C).

Finally, it has previously been shown that the degradation of amyloid fibrils in Alzheimer's disease requires the delivery of ClC-7 to the lysosomes (Lee et al., 2020; Majumdar et al., 2011), and inefficient degradation of amyloid plaques by microglia in Alzheimer's disease is at least in part due to abnormal lysosomal acidification (Lee et al., 2020; Majumdar et al., 2007). To test the ability of microglia to effectively degrade the amyloid peptide *in vivo*, we injected β-amyloid (1–42)–HiLyte Fluor 555 into the midbrain of zebrafish larvae. We found an increased accumulation of β-amyloid particles within the microglia of *Clcn7* mutants compared to heterozygotes (Fig. 4C). Together, our observations demonstrate that cells of the macrophage lineage exhibit a reduced capacity to degrade endogenous as well as exogenous substrates. Future experiments will help illuminate whether the accumulation of debris inside microglia and macrophages in *clcn7* mutants is due to a complete or partial loss of degradative function and explore mechanisms through which the clearance capabilities can be restored.

The molecular mechanism through which ClC-7 and Cl^−^ levels regulate the activity of lysosomes remains unclear. Although the prevailing model suggests that ClC-7 is a Cl^−^–H^+^ exchanger (Graves et al., 2008; Ishida et al., 2013; Leray et al., 2022; Mindell, 2012; Weinert et al., 2010), multiple lines of evidence indicate that lysosomal pH is normal in *Clcn7* as well as *Ostm1* mutants (Jentsch and Pusch, 2018; Kasper et al., 2005; Kornak et al., 2001). Furthermore, whereas disruption of lysosomal pH has been extensively reported in several mammalian cell culture models of lysosomal storage disorders (Bach et al., 1999; Holopainen et al., 2001; Sillence, 2013), recent evidence indicates that the drop in Cl^−^ concentration in these diseases may be significantly greater relative to the concomitant decrease in H^+^ concentrations (Chakraborty et al., 2017). These observations, along with ours, further underscore the importance of Cl^−^ transport for lysosomal function even though the lumenal pH of lysosomes might be independent of Cl^−^ transport in at least some instances.

### Conclusions

The surveillance, clearance and phagocytic capabilities of microglia are intimately linked to the appropriate activation and function of lysosomes. Multiple ion channels on the lysosomal membrane act in concert with one another to achieve a unique ionic microenvironment inside lysosomes (Kendall and Holian, 2021; Kettenmann et al., 2011). The delicate balance of ions within lysosomes is in turn crucial for the regulation of the pH, proteolytic activity and processing capacity of these critical organelles. Here, we show that the chloride transporter, ClC-7, and its partner Ostm1, are essential for the normal function of microglia. In zebrafish *clcn7* mutants*,* we find that microglia are unable to degrade extracellular debris effectively. Our findings are concordant with recent studies in *Caenorhabditis elegans* showing that a reduction in Cl^−^ levels correlates with a diminished degradative capacity of lysosomes (Chakraborty et al., 2017). Our data highlight the importance of Cl^−^ channel activity for the normal function of microglia. Our observations also have important implications for understanding the role of microglia in neurodegenerative phenotypes that accompany lysosomal storage disorders and autosomal recessive osteopetrosis.

## MATERIALS AND METHODS

### Zebrafish lines

Embryos from wild-type (TL or AB; stocks maintained at Stanford University) strains and *Tg(mpeg:GFP)* (Ellett et al., 2011) were raised at 28.5°C. For all imaging experiments (except where noted), embryos were treated with 0.003% 1-phenyl-2-thiourea (PTU) in Methylene Blue embryo water (The Zebrafish Book; https://zfin.org/zf_info/zfbook/zfbk.html) to inhibit pigmentation, and anesthetization was performed using 0.016% MS-222 (Tricaine) prior to experimental procedures. All animal protocols have been approved by the Stanford Institutional Animal Care and Use Committee.

### CRISPR injections

sgRNAs targeting *clcn7* and *ostm1* were designed using CHOPCHOP (https://chopchop.cbu.uib.no/). Oligonucleotides containing the T7-binding site and the CRISPR sequence were annealed to tracrRNA template. Assembled oligonucleotides were transcribed using HiScribe T7 Quick (NEB, E2050S) kit. Following DNase treatment, the RNA was purified using mirVana miRNA isolation kit (Invitrogen, AM1561). An aliquot of the sgRNA eluate was run on agarose gel and quantified using nanodrop. CRISPR injections were performed at the one-cell stage. The injection mix consisted of 300 ng/µl of Cas9 protein and 300 ng/µl of the sgRNA in Tris-HCl pH 7.5. A small amount of Phenol Red was added to the mix to help with visualization during injection. The F1 progeny of F0-injected fish were verified for an out-of-frame insertion or deletion. Details of guideRNA and primers used: *clcn7* sgRNA sequence 5′-GGAGCGGTGTTCTCTCTCCGCGG-3′; *clcn7* forward primer 5′-ACGGAAGTGCAACCCACAAC-3′; *clcn7* reverse primer 5′-GGTCACGGGATTGCTTATGATTG-3′; *clcn7* sgRNAs used for determining off-target effects: 5′-GGAGAACACCGCTGCTGAACGG-3′, 5′-GGCTGCGGGAGTTTCTCAAGG-3′ and 5′-GGCGGGAGTTTCTCAAGGCAGG-3′. All three guide RNAs were co-injected with Cas9 to examine possible off-target effects in Figs S2 and S4. *ostm1* sgRNA sequence 5′-GGTCTTCTATATCAATGCAGAGG-3′; *ostm1* forward primer 5′-CTCTGCAAGGACTGCAAACCC-3′; *ostm1* reverse primer 5′-CAGCTCTTTGGTGCTTACTTTGG-3′.

### Neutral Red assay

Neutral Red (Sigma, N4638) was dissolved in distilled water to make a 2.5 mg/ml stock solution, which is stable at room temperature for several months. Staining using Neutral Red was undertaken by treating larvae at 4 days post fertilization (dpf) with 5 µg/ml solution of Neutral Red in embryo water containing PTU for 3 h. Animals were washed at least twice after 3 h of incubation and left overnight in embryo water with PTU at 28.5°C to wash out the dye. Anesthetization and mounting in 1.5% agarose were undertaken at ∼24 h after Neutral Red treatment. The number of Neutral Red-positive microglia was counted, and the image was acquired immediately after counting using Zeiss AxioCam HRc camera with the AxioVision software. All the genotyping and statistical analysis were done post imaging.

### *In situ* hybridization

*apoe* and *mbp* antisense probes were synthesized as previously described (Lyons et al., 2005; Shiau et al., 2013). *In situ* hybridization was performed using standard methods (Thisse and Thisse, 2008). Briefly, embryos at 4 dpf were fixed overnight in 4% paraformaldehyde, dehydrated overnight in 100% methanol, rehydrated in PBS containing Triton X-100, and then PBS alone, permeabilized using proteinase K at a dilution of 1:1000 for 1 h, incubated overnight with antisense riboprobes at 65°C. Following washes in 2× and 0.2× SSC, and incubation in MAB block containing 10% normal sheep serum, animals were incubated overnight at 4°C in block solution containing 1:1000 dilution of anti-digoxigenin antibody conjugated to alkaline phosphatase. The following day, after at least six 20-min washes in MAB-Triton X-100 buffer, animals were incubated in the development solution containing NBT (Roche, 11383213001) and BCIP (Roche, 11383221001). Development was stopped at the same time for all samples in a single experiment by evaluating the strength of the signal in control animals. Animals were washed twice in PBS with Triton X-100, left in 100% ethanol overnight for destaining, and rehydrated in PBS with Triton X-100. Animals were mounted in 100% glycerol and images were captured using Zeiss AxioCam HRc camera with the AxioVision software. All the genotyping and statistical analysis were done post imaging.

### Transgene constructs and injection

Plasmids expressing the transgenic constructs *Tg(mpeg:mCherry-Rab7; cmcl2:GFP)* or *Tg(mpeg:LAMP1b-mCherry; cmlc2:GFP)* were co-injected at 12–25 pg along with 50–100 pg of Tol2 transposase mRNA at one-cell stage (Iyer et al., 2022). *Tg(cmlc2:GFP)* was used as the selection marker for mCherry–Rab7 and LAMP1b-mCherry fusion constructs and imaging was performed following confirmation of *Tg(cmlc2:GFP)* expression at 4 dpf. All the genotyping and statistical analysis were done post imaging.

### LysoTracker Red assay

LysoTracker Red DND-99 (Thermo Fisher Scientific, L7528) was dissolved in embryo water with PTU at 1:100 dilution. Larvae at 4 dpf were incubated in the LysoTracker Red solution for 30–45 min. After staining, larvae were washed in embryo water containing PTU for 30 min, with at least three washes ∼8–10 min apart. Following anesthetization and mounting, images were acquired using Zeiss LSM confocal microscope. The same imaging parameters (laser power, zoom and gain etc.) were used for a single experiment, all genotyping was done post imaging. The area and intensity of LysoTracker Red punctae were calculated using ImageJ.

### TUNEL assay

Larvae were fixed at 4 dpf with 4% paraformaldehyde for 2 h at room temperature. Fixed larvae were permeabilized using proteinase K (Thermo Fisher Scientific, 25530049) at a dilution of 1:1000 for 30 min and post-fixed using 4% paraformaldehyde for 20 min at room temperature. Larvae were incubated in blocking solution (1% DMSO, 1% donkey serum, 1% BSA, 0.7% Triton X-100 in 1× PBS) for 2 h at room temperature and incubated in blocking solution with anti-GFP antibody (1:500, Abcam, ab6658) overnight at 4°C. Following six 10-min washes in 1× PBS with 0.8% Triton X-100, larvae were incubated in blocking solution with 1:500 donkey anti-goat-IgG conjugated to Alexa Fluor 488 or 594 for 2 h at room temperature, followed by six 10-min washes in 1× PBS with 0.8% Triton X-100. Larvae were incubated in 1:10 dilution of *in situ* cell death detection kit solution (Roche, SKU 12156792910) (10 µl enzyme solution with 90 µl label solution) and incubated at 37°C in the dark for 2 h. Following washes with 1× PBS with 0.8% Triton X-100 overnight, animals were mounted in 2% agarose and imaged. Following imaging, the intensity of TUNEL punctae inside microglia was calculated using ImageJ. Genotyping and statistical analysis were done post imaging.

### Acridine Orange assay

Zebrafish larvae at 4 dpf were incubated in 5 µg/ml Acridine Orange (Sigma A9231) solution for 45 min. Larvae were washed in embryo water containing PTU for 45 min, with at least the washes ∼10–15 min apart. Following anesthetization, all larvae were mounted laterally, and images were acquired using Zeiss LSM confocal microscope. The number of AO^+^ punctae in the head, eyes and tail were counted and all genotyping was done post imaging.

### Tail fin injury assay

Animals at 4 dpf were anesthetized and the tip of the tail fin was cut using a new scalpel ensuring approximately the same size of incision in all animals. Larvae were allowed to recover in embryo water. At 12 h post injury, animals were mounted in 1.5% agarose with anesthetic. The number of macrophages at the site of injury (regenerated fin tissue and blastema) was counted and imaging was undertaken using a 20× objective using Zeiss LSM Confocal microscope. Genotyping and statistical analysis were done post imaging.

### Neuronal debris quantitation

Larvae expressing *NBT:DsRed* and *mpeg:GFP* transgenes were imaged with 10× and 20× objectives using a Zeiss LSM confocal microscope. Following imaging, the intensity of DsRed punctae inside microglia in the midline of the animal was calculated using ImageJ. All the genotyping and statistical analysis were done post imaging.

### *E. coli* Texas Red injection and imaging

*E. coli* Texas Red (Thermo Fisher Scientific E2863, 20 mg/ml in 1× PBS) injection was performed at 4 dpf by injecting 1 nl of the microbial suspension into the yolk of anesthetized larvae (Benard et al., 2012). Larvae were allowed to recover for 1 h, anesthetized, mounted in 1.5% agarose and imaged at 4 dpf using a Zeiss LSM confocal microscope. Following imaging, the intensity of Texas Red punctae inside the tail macrophages was calculated using ImageJ. All the genotyping and statistical analysis were done post imaging.

### Zymosan A injection and imaging

Zymosan A (Sigma A4250, 1% in 1× PBS) injection was performed at 5 dpf by injecting 1 nl of the solution into the ventricles of the midbrain or optic tectum. Larvae were incubated for 2 h, anesthetized, mounted in 1.5% agarose and imaged using a Zeiss LSM confocal microscope. Non-injected controls were incubated under the same conditions. All the genotyping and statistical analysis were done post imaging.

### β-amyloid (1–42) injection and imaging

β-amyloid (1–42) HiLyte-Fluor555 (Anaspec, AS-60480-01) was reconstituted by adding 100 µl of 1% NH_4_OH to 1 mg β-amyloid and the stock was diluted to 1 mg/ml in 1× PBS. Injections were performed at 5 dpf by injecting 1 nl of the β-amyloid suspension into each hemisphere of the midbrain of anesthetized larvae. Larvae were allowed to recover for 24 h, anesthetized, mounted in 1.5% agarose and imaged at 6 dpf using a Zeiss LSM confocal microscope. Following imaging, the intensity of Fluor-555 punctae inside the microglia was calculated using ImageJ. All the genotyping and statistical analysis were done post imaging.

## Supplementary Material

10.1242/joces.261616_sup1 Supplementary information
