# Supplementary material for: The Cl− transporter ClC-7 is essential for phagocytic clearance by microglia
Source: J Cell Sci. 2024 Feb 16;137(4):jcs261616. doi: 10.1242/jcs.261616 (PMC10911276; doi:10.1242/jcs.261616)
Supplement: Supplementary information [file joces-137-261616-s1.pdf]

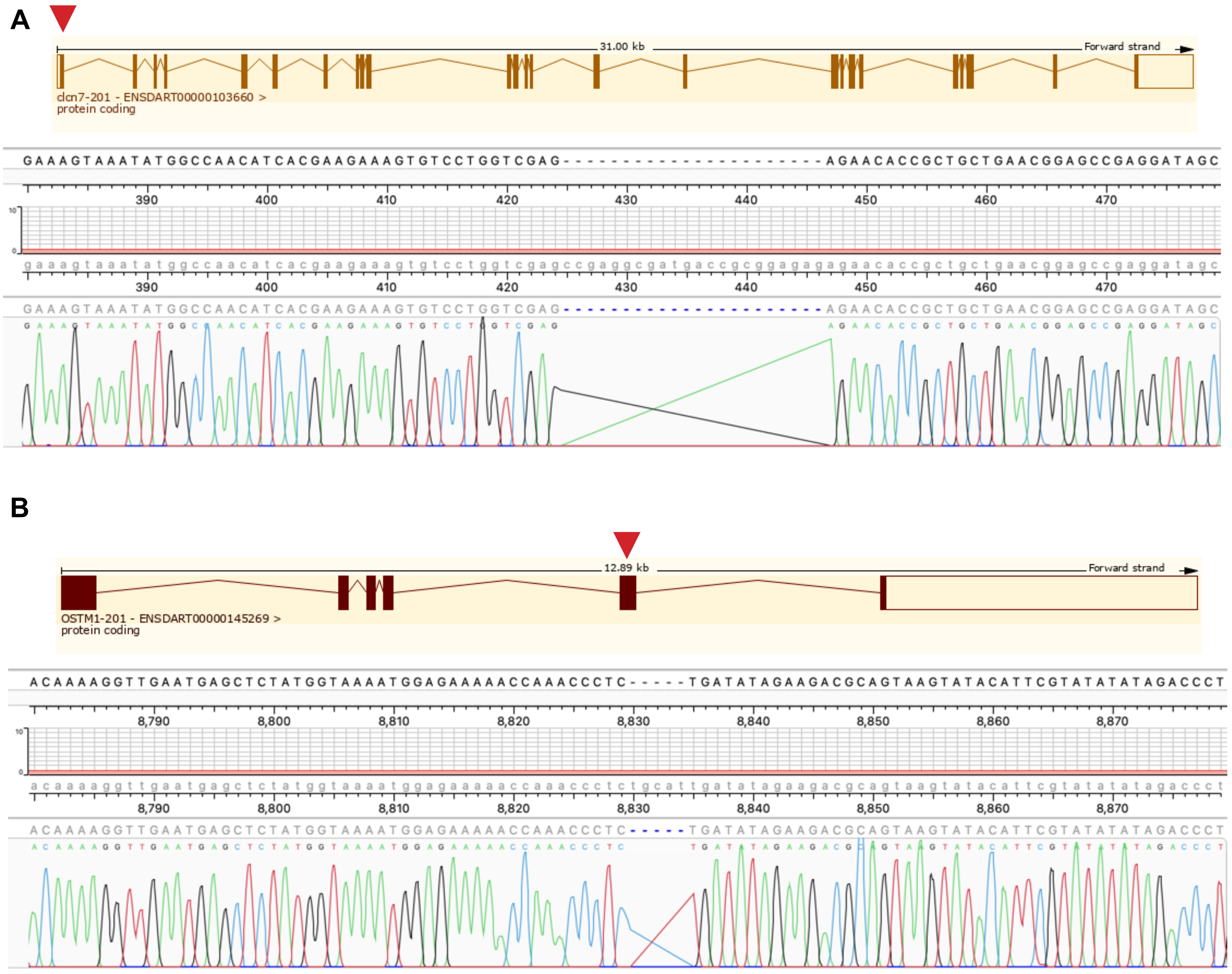

Fig. S1. Chromatograms showing the mutations in *clcn7* (st167) and *ostm1* (st168). A. *clcn7* mutants have a 22 bp deletion in exon 1 (arrowhead on gene structure at top). **B.** *ostm1* mutants have a 5 bp deletion in exon 5 (arrowhead on gene structure at top). These deletions introduce frameshift mutations and truncate the predicted proteins.

Journal of Cell Science • Supplementary information

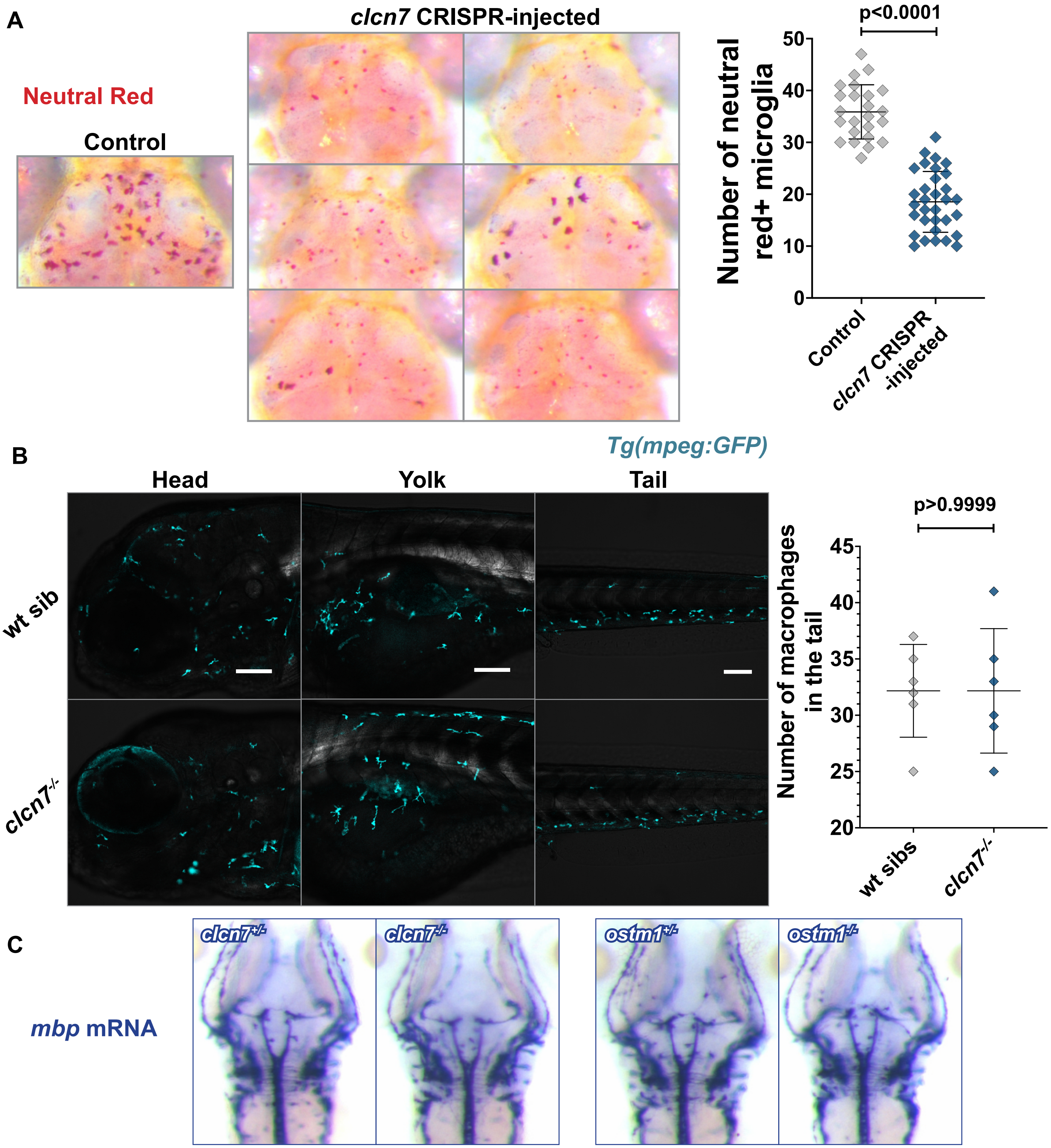

Journal of Cell Science • Supplementary information

Fig. S2. A. Synthetic guide RNAs targeting different regions of the *clcn7* genomic locus were injected into one-cell zebrafish embryo, followed by neutral red staining at 4 days post fertilization. (Control: N=24, mean=35.9, SD=5.2, SEM=1.1. *clcn7* CRISPR-injected: N=32, mean=18.5, SD=5.9, SEM=1.0.) **B.** *mpeg1:GFP* expression showing that numbers of macrophages in the head, yolk, and tail regions of *clcn7* mutants are similar to wildtype siblings at 4 dpf. Scale bars, 100 µm. Tail macrophages: wildtype siblings N=6, mean=32.2, SD=4.1, SEM=1.7; *clcn7* mutants N=6, mean=32.2, SD=5.5, SEM=2.3. **A, B.** Two-tailed unpaired t-test with Welch's correction was performed to determine statistical significance; graph shows mean with SD. **C.** in situ hybridization showed similar expression of *mbp* in *clcn7* and *ostm1* heterozygotes and mutants at 5 dpf.

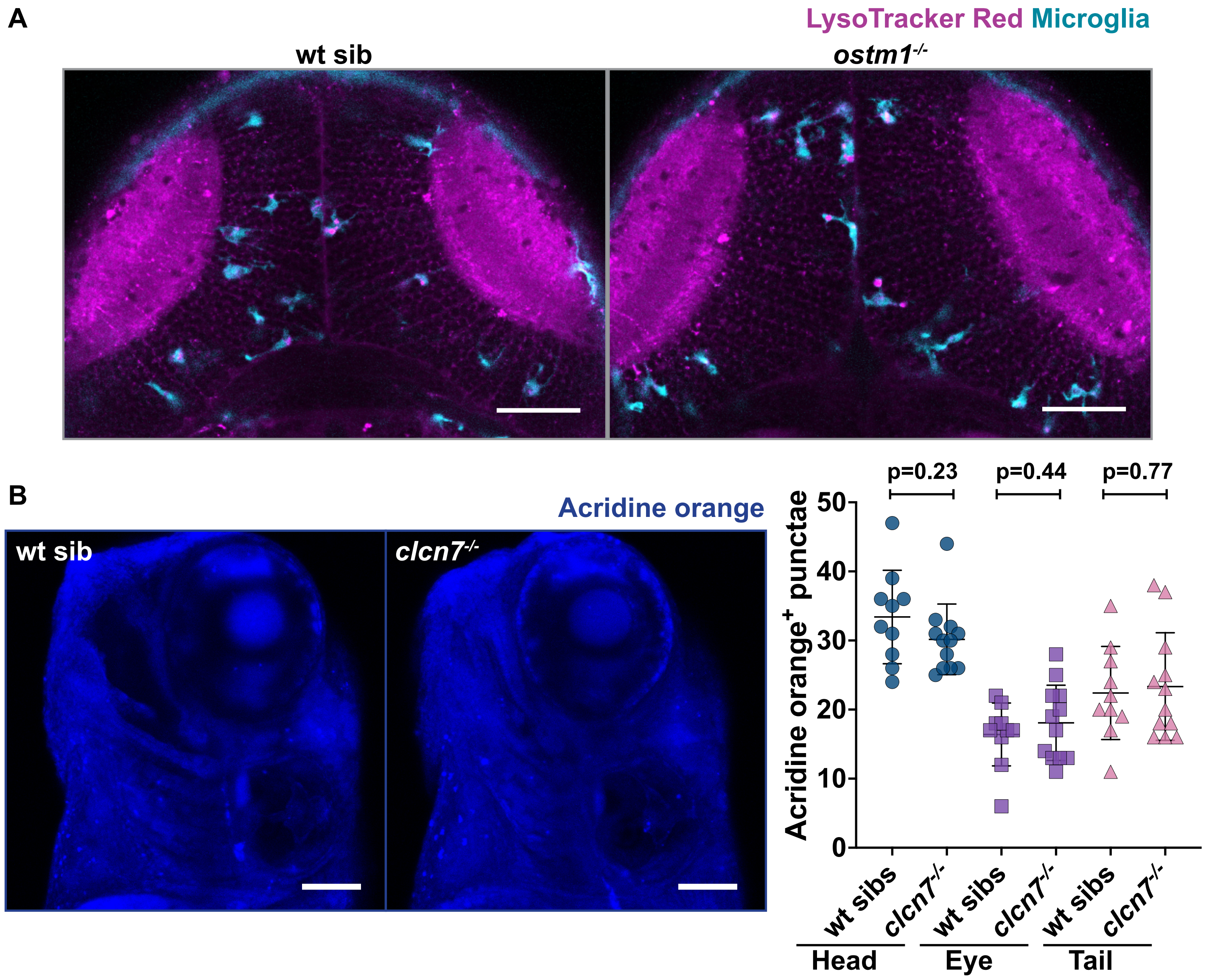

Fig. S3. A. LysoTracker Red labeling is comparable in *ostm1* mutants and wildtype siblings. **B.** Acridine orange labeling shows that the number of apoptotic cells were similar in *clcn7* mutants and their wildtype siblings in the head (wt sibs: N=10, mean=33.4, SD=6.8, SEM=2.1 and mutants: N=12, mean=30.2, SD=5.1, SEM=1.5), eye (wt sibs: N=10, mean=16.4, SD=4.6, SEM=1.4 and mutants: N=12, mean=18.1, SD=5.5, SEM=1.6), and tail (wt sibs: N=10, mean=22.4, SD=6.7, SEM=2.1 and mutants: N=12, mean=23.3, SD=7.8, SEM=2.3). All counts are from lateral mounts. Two-tailed unpaired t-test with Welch's correction was performed to calculate significance; graphs show mean with SD. All scale bars, 100 µm.

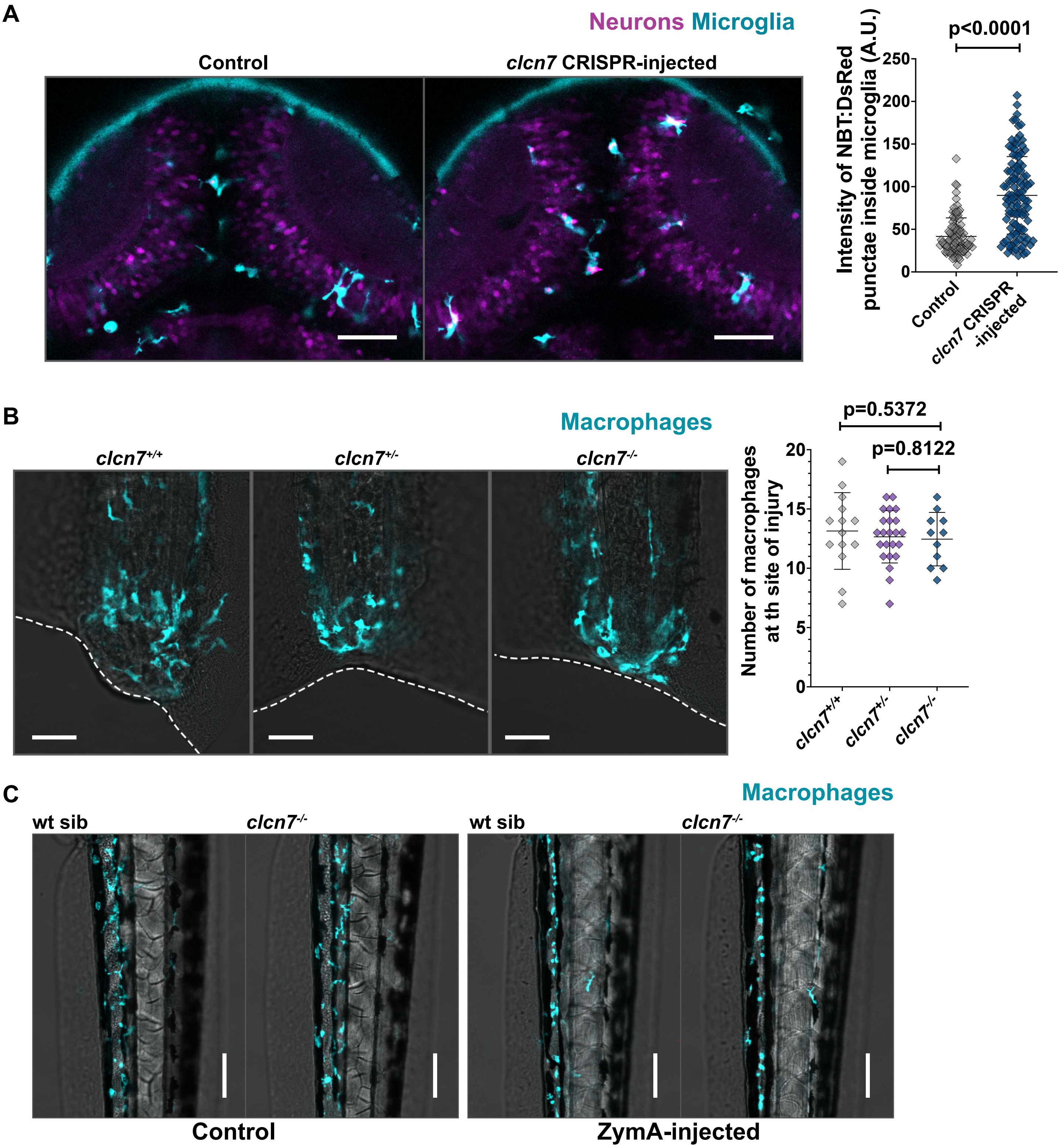

Journal of Cell Science • Supplementary information

Fig. S4. A. Mosaic animals injected with *clcn7* CRISPRs targeting different genomic loci also show defects in neuronal debris clearance (control: N=33, mean=41.7, SD=21.7, SEM=2.2 and *clcn7* CRISPants: N=34, mean=89.8, SD=45.5, SEM=4.3). Scale bars, 50 μm. **B.** Embryonic macrophages in *clcn7* mutants effectively respond to peripheral injury effectively (*clcn7*$^{+/+}$: N=14, mean=13.1, SD=3.2, SEM=0.9. *clcn7*$^{+/-}$: N=23, mean=12.7, SD=2.2, SEM=0.5. *clcn7*$^{-/-}$: N=11; mean=12.5, SD=2.3, SEM=0.7). Scale bars, 50 μm. Two-tailed unpaired t-test with Welch's correction was performed to calculate significance; graphs show mean with SD. **C.** Macrophages in *clcn7* mutant embryos not treated with PTU and injected with ZymA respond similarly to macrophages in wildtype siblings. Scale bars, 100 μm.
